# Ureteric stent versus percutaneous nephrostomy for acute ureteral obstruction - clinical outcome and quality of life: a bi-center prospective study

**DOI:** 10.1186/s12894-019-0510-4

**Published:** 2019-08-28

**Authors:** O. Shoshany, T. Erlich, S. Golan, N. Kleinmann, J. Baniel, B. Rosenzweig, A. Eisner, Y. Mor, J. Ramon, H. Winkler, D. Lifshitz

**Affiliations:** 1 Urology Department, Rabin Medical Center, Hasharon Hospital, 7 Keren Kayemet St, 49372 Petah Tikva, Israel; 2 Sackler School of medicine, Tel Aviv University, Tel Aviv, Israel; 3 Urology Department, Chaim Sheba Medical Center, Ramat Gan, Israel

**Keywords:** Double J stent, Nephrostomy tube, Acute ureteral obstruction, Quality of life

## Abstract

**Background:**

To compare quality of life (QoL) indices between ureteral stent (DJS) and nephrostomy tube (PCN) inserted in the setting of acute ureteral obstruction.

**Methods:**

Prospective bi-centered study. Over the span of 2 years, 45 DJS and 30 PCN patients were recruited. Quality of life was assessed by 2 questionnaires, EuroQol EQ-5D and ‘Tube symptoms’ questionnaire, at 2 time points (at discharge after drainage and before definitive treatment).

**Results:**

Patients’ demographics and pre-drainage data were similar. There were no clinically significant differences in patient’s recovery between the groups, including post procedural pain, defeverence, returning to baseline renal function, and septic shock complications.

More DJS patients presented to the emergency room with complaints related to their procedure compared to PCN patients. At first, DJS patients complained more of urinary discomfort while PCN patients had worse symptoms relating to mobility and personal hygiene, with both groups achieving similar overall QoL score. At second time point, PCN patients’ symptoms ameliorated while symptoms in the DJS group remained similar, translating to higher overall QoL score in the PCN group.

**Conclusions:**

The two techniques had a distinct and significantly different impact on quality of life. Over time, PCN patients’ symptoms relieve and their QoL improve, while DJS patients’ symptoms persist. Specific tube related symptoms, and their dynamics over time, should be a major determinant in choosing the appropriate drainage method, especially when definitive treatment is not imminent.

**Electronic supplementary material:**

The online version of this article (10.1186/s12894-019-0510-4) contains supplementary material, which is available to authorized users.

## Background

The decision which drainage method, when indicated, should be used in a patient presenting with an obstructing ureteral stone is significantly influenced by the clinician’s and patient’s perception of the risks, complexity and quality of life (QoL) issues related to the different drainage methods. In certain health systems, such as the public health system in Israel, the waiting time for stone removal surgery is long. We often observed that an expected long period time for definitive treatment biased the treating urologist to prefer a ureteral stent over a nephrostomy tube, assuming the patient would be less bothered by the stent during the waiting period. There are few studies comparing the efficacy of ureteral double J stent (DJS) vs a percutaneous nephrostomy tube (PCN) in the setting of obstructive ureteral stones. Two prospective studies [1, 2], both published in 2001, incorporated QoL questionnaires and compared patients’ perception of the two drainage methods while awaiting definitive stone treatment. The results of these studies are not conclusive regarding the gross impact of each drainage method on the patient’s QoL. The study by Joshi et al. [1] was conducted at a single time point, upon patients’ admission for lithotripsy. No significant difference was found in the overall health state, suggesting similar patient’s preference for either modality treatment. Conversely, in the study by Mokhmalji et al. [2] QoL was assessed at two time points, immediately following drainage and 2–4 weeks thereafter. Although not statistically significant, a tendency in favor of PCN was evident, particularly in males and patients younger than 40. The QoL progressively improved in the PCN group but deteriorated in the DJS group. Another recent prospective non-randomized study [3] evaluated QoL before and after the drainage procedure. DJS patients demonstrated worse QoL after drainage while PCN patients had similar ratings. DJS patients had worse urinary symptoms and a higher need of painkillers as well. Likewise, in a prospective study, albeit in a different setting, comparing nephrostomy vs ureteral stent following percutaneous nephrolithotomy (PCNL), Zhao et al. [4] found that despite the literature advocating “tubeless” PCNL, the QoL is significantly worse with stent placement in comparison to nephrostomy drainage. Similarly, Jiang et al. [5] compared PCN, an open-ended ureteral catheter and a DJS following PCNL, and found that patients in the first two groups reported a better QoL. Contrarily, in the setting of malignant obstruction, similar QoL was achieved in both PCN and DJS groups [6].

The aim of the present study was to evaluate whether these two renal drainage techniques truly have similar impact on the patient’s QoL, in the setting of acute ureteral obstruction, and whether their effect changes over time during the waiting period for definitive treatment. Such data is important for the shared decision between the physician and the patient as to the preferred drainage technique.

## Methods

Over a 2 year period, 75 consecutive patients presenting to the emergency room of two hospitals with an obstructing ureteral stone necessitating drainage were asked to participate in this study by an on-call urologist involved in the study. Estimated recruitment rate was 42%. The study is an Institutional Review Board approved, bi-center, prospective study comparing the impact on QoL of PCN and retrograde ureteral catheterization. Patients were offered renal drainage when definitive therapy was not available immediately or was contraindicated and when a two-stage procedure was considered a safer approach. Entry criteria included an obstructing ureteral stone with either fever (> 38°c), acute renal failure (estimated glomerular filtration rate [eGFR] ≤ 60 ml/min) or intractable pain. Diagnosis of the obstructing ureteral stone was made by either a non-contrast CT or a combination of renal ultrasound and abdominal X-ray. Exclusion criteria were age < 18 years, pregnant women and patients with a contraindications to either form of drainage (e.g. uncorrected coagulopathy excluding percutaneous drainage, hemodynamic instability precluding anesthesia required for DJS placement, or abnormalities of the urinary tract). Patients received either a DJS or a PCN according to the surgeon’s preference. Practical issues such as the availability of an operating room or the interventional radiologist often dictated the choice of drainage, particularly in patients with fever as it is our policy to reduce time to drainage to the minimum in this high risk group.

PCN was performed in the angiographic suite by certified interventional radiologists. Local anesthesia (1% Lidocaine, 5–10 cc) was used routinely and when required additional parenteral analgesia with Meperidine (50-75 mg) and Midazolam (3 mg) was administrated. A percutaneous pigtail polyurethane 8.5-french, 25 cm catheter (Cook medical) was used. Ureteral stents were placed by the urologist in the operating room under general anesthesia. Initially, a guide wire was inserted into the kidney followed by a ureteral 5FR catheter. A urine culture was taken and contrast was injected at the same volume that was withdrawn for urine culture. Finally, a 6 FR, Percuflex (Boston Scientific) stent of the appropriate length was used. Most commonly, stent length of 24 to 28 cm was utilized. The ureteral length between the ureteropelvic and ureterovesical junctions was determined either by preoperative CT or by intraoperative retrograde and ureteral catheter placement.

Demographic and preoperative data were obtained included age, gender, body mass index (BMI), baseline and post drainage eGFR, urine culture and stone parameters. Post-procedural pain was measured on the day of the procedure, using a verbal visual analogue scale (VAS). QoL was assessed twice: at post-operative day 1–2 (“time 0”) and at the day of (prior to) definitive treatment (“time 1”). Patients were requested to fill out two dedicated questionnaires: EuroQol EQ-5D and a ‘Tube symptoms’ questionnaire. EuroQol EQ-5D is a validated tool of general health assessment, consisting of 5 QoL questions and a thermometer indicating general well-being [1, 7]. A higher questionnaire score is associated with lower QoL, while a higher thermometer score is associated with better QoL. The ‘Tube symptoms’ questionnaire was based on several of the questions in the “Intervention-specific” questionnaire described by Joshi [8] with some modifications to allow uniformity of the questions between the groups. For example, patients were not asked about ‘urine incontinence’ or ‘Bag leak/slippage’. Alternatively, they were asked about ‘personal hygiene problems’ due to indwelling tube. The modified questionnaire includes six questions regarding pain, analgesics use, hematuria, urinary discomfort, discomfort associated with movement, and discomfort associated with personal hygiene. Answers to these questions were based on a four-point rating scale, and a higher score is associated with worse symptoms (see Additional file 1).

### Statistical analysis

The association between different groups was assessed using the chi-square, 2-tailed Student t test and Mann-Whitney U tests, as appropriate. All statistical tests were 2-sided and for all comparisons *P* < 0.05 was considered significant. Statistical analysis was done using SPSS®, version 20.0.

## Results

### Pretreatment parameters

Overall, 45 patients underwent a DJS insertion and 30 patients had PCN drainage. Patients’ demographics and pre-drainage data are presented in Table 1. Pretreatment differences in age, male to female ratio, and BMI were not significant. Stone parameters were also comparable, and the proportion of stone location (either proximal or distal ureter) was similar between the groups (*p* > 0.05). There were no cases of bilateral treatment. The indications for drainage were similar between the groups. Patients presenting with acute renal failure amounted to 57.8% in the DJS group and 60% in the PCN group. The only significant difference between the groups was pre-drainage eGFR: patients in the PCN group were found to have lower baseline eGFR and lower eGFR at presentation. Patients with fever had median temperatures of 38.1°c (range 38–39.5 °c) and 38.6 °c (range 38–39.6 °c) in the DJS and PCN groups, respectively.

**Table 1:** Patients’ characteristics and outcomes

	DJS (*n* = 45)	PCN (*n* = 30)	*p* value
Pre-drainage patients’ characteristics			
Age (years)	55 (39.5–70.5)	54 (46.5–61)	0.787
Gender- Female	15 (33%)	13 (43%)	0.467
BMI (kg/m^2^)	27.6 (24.8–30.1)	27.7 (23.3–31)	0.944
Hypertension	24 (53.3%)	13 (43.3%)	0.683
Diabetes mellitus	14 (31.1%)	7 (23.3%)	0.622
Ischemic heart disease	8 (17.7%)	7 (23.3%)	0.775
Previous endourological procedures	14 (31.1%)	9 (30%)	1
Baseline eGFR (MDRD, mL/min/1.73 m^2^)	85.5 (69.3–90.6)	69 (58.1–80.4)	**0.001**
eGFR at presentation (MDRD, mL/min/1.73 m^2^)	60.3 (41.3–75.6)	41.2 (33–59)	**0.011**
Positive urine cultures	25.6%	40.7%	0.199
Indication for drainage – Fever≥30^o^c	15 (33.3%)	16 (53.3%)	0.099
Indication for drainage - Renal Failure (eGFR≤60 mL/min/1.73 m^2^)	10 (22.2%)	10 (33.3%)	0.301
Stone diameter (mm)	8 (7–11)	8 (6–12.3)	0.872
Stone location-Proximal	55%	64%	0.469
Stone location-Distal	45%	36%	0.469
Post drainage outcomes			
Post procedure hospitalization Days	1 (1–3)	4 (2–6)	**< 0.001**
Post Procedural Pain (VAS)	1.02 ± 2.04	1.19 ± 1.52	0.283
Days to baseline eGFR ^@^	1 (1–2)	2 (1–3)	**0.005**
Days to Temp ≤37.5 ^@@^	1 (1–1.5)	1 (1–1.75)	1
Time to WBC ≤10,000 white blood cells per microliter (Days) ^@@@^	1.5 (1–2)	2 (1–4)	0.167
Complications 1st procedure	6 (11%)	1 (3.3%)	0.226
Time from 1st to 2nd operation (Days)	47 (29–71)	20 (12–27)	**< 0.001**

Data presented as Median (IQR 25–75) or Mean ± STD as appropriate

*MDRD* The Modification of Diet in Renal Disease Study equation

^@^ For patients with renal failure at presentation

^@@^ For patients with fever at presentation

^@@@^ For patients with leukocytosis at presentation

In bold - statistically significant result

### Post-treatment parameters

Post renal drainage outcomes are presented in Table 1. Post procedural pain was similar in both groups as measured with VAS. The median length to defeverence was 1 day in both groups, while the median length of white blood count (WBC) normalization was 1.5 and 2 days, respectively. Three patients in the DJS group and one patient in the PCN group developed post drainage septic shock (*p* = 0.646). The median time to baseline eGFR level was found to be significantly higher in the PCN group (1 and 2 days respectively, *p* < 0.01). After discharge, none of the patients required readmission to the hospital. Although not statistically significant, more DJS patients presented to the emergency room with complaints related to their procedure compared to PCN patients (20% vs. 3.7% respectively, *p* = 0.056), mostly complaining of flank pain and dysuria. During the study period there were no cases of failed procedures or conversion from one technique to the other, and post-procedural complication rate was similar between the groups. The majority of patients in both groups underwent ureteroscopy as the definitive procedure. The length of time between the urgent drainage procedure and the definitive procedure was significantly higher in the DJS group compared to the PCN group (median 47 vs. 20 days respectively, *p* < 0.001).

### Quality of life results

#### ‘Tube symptoms’ questionnaire

Table 2 details the patients’ scores in the ‘Tube symptoms’ questionnaire at time0 (shortly after the drainage procedure) and time1 (presenting for their definitive procedure). For each symptom, data is presented as percentage of patients reporting the presence of the symptom and an estimation of the frequency of the symptom using a 4 points scale. At both time points, the two groups reported similar pain, analgesics use, and presence of hematuria. However, other domains were remarkably different between the two groups. At time0, DJS patients had significantly higher urinary discomfort. Symptoms in this domain were much more prevalent and frequent compared to PCN patients. Pronounced urinary discomfort persisted in the DJS group, remaining significantly higher than the PCN group at time1. On the other hand, PCN patients had worse symptoms relating to mobility and personal hygiene. However, the severity of these symptoms decreased over time, and at time1 there were no significant differences between the groups in these domains. Interestingly, women reported using pain killers more frequently at time0 in both groups (*p* = 0.022 and *p* = 0.04 for DJS & PCN respectively), but not at time1.

**Table 2:** QoL outcomes - ‘Tube symptoms’ questionnaire

	At discharge after urgent drainage						Upon arrival for definitive stone treatment					
	DJS		PCN		p value		DJS		PCN		*p* value	
Q1 Pain	45.5%	1.73 ± 0.94	48.3%	1.83 ± 1	1	0.755	76.7%	2.13 ± 0.9	50%	1.73 ± 0.88	0.076	0.082
Q2 Need of analgesia	40.1%	1.55 ± 0.74	62.1%	1.86 ± 0.79	0.164	0.138	50%	1.8 ± 1	40.1%	1.59 ± 0.85	0.581	0.461
Q3 Urinary discomfort	80.1%	2.71 ± 1.1	31%	1.59 ± 0.98	**0.0006**	**< 0.001**	80.6%	2.55 ± 1.06	45.5%	1.91 ± 1.15	**0.017**	**0.034**
Q4 Hematuria	54.5%	2 ± 1.15	37.9%	1.62 ± 0.9	0.268	0.216	48.4%	1.87 ± 1.02	27.3%	1.45 ± 0.91	0.159	0.104
Q5 Mobility	42.9%	1.71 ± 0.96	86.2%	2.66 ± 1.04	**0.0019**	**0.002**	32.3%	1.55 ± 0.89	45.5%	1.82 ± 1.05	0.395	0.317
Q6 Personal hygiene	33.3%	1.43 ± 0.68	60.7%	2.18 ± 1.09	0.084	**0.014**	25.8%	1.35 ± 0.66	40.1%	1.77 ± 1.07	0.371	0.155

Data presented as percentage of symptomatic patients (reporting any symptoms) and frequency score as reported using a 4 points scale

In bold - statistically significant result

#### EQ-5D questionnaire

EQ-5D questionnaire outcomes are presented in Table 3. At time0, PCN patients demonstrated more difficulties to self-care and resume usual activities (*p* < 0.05). Twice as many PCN patients reported difficulties in mobility compared to DJS patients, which was in accordance with the ‘Tube symptoms’ questionnaire results, but this did not reach statistical significance (16.6% vs. 34.5% for the DJS and PCN groups respectively, *p* > 0.05). In league with the trend observed in the ‘Tube symptoms’ questionnaire, symptoms which were higher in PCN patients decreased over time, and at time1 we observed no significant difference in patients’ estimation of their ability to self-care or perform usual activities. Additionally, more DJS patients reported anxiety or depressed mood compared to PCN patients (19.4% vs. 0%, *p* < 0.05). It is important to note that both procedures caused pain or discomfort to a large portion of patients in both groups at both time points: 47.6% vs. 53.6% at time0 and 67.7% vs. 42.9% at time1, respectively. These proportions were not significantly different, although there were opposing trends in the different groups: over time the number of patients complaining about pain increased in the DJS-treated group, and decreased in the PCN-treated group. While in the PCN group there were no differences between the genders, in the DJS group women scored worse in the self-care domain at time0 (*p* = 0.025), and in pain / discomfort domain at both time points (*p* = 0.049 & *p* = 0.048 at time0 & time1, respectively).

**Table 3:** QoL outcomes - EQ-5D questionnaire

	At discharge after urgent drainage			At definitive stone treatment		
	DJS	PCN	p value	DJS	PCN	*p* value
Q1 Mobility	16.60%	34.50%	0.102	25.80%	18.20%	0.51
Q2 Self-care	7.10%	37.90%	**0.004**	9.70%	9.10%	0.944
Q3 Usual activities	19%	57.70%	**0.005**	38.70%	28.60%	0.39
Q4 Pain/discomfort	47.60%	53.60%	0.632	67.70%	42.90%	0.082
Q5 Anxiety/depression	35.70%	20.70%	0.199	19.40%	0%	**0.012**
“Health state” score	72.6 ± 22	68.6 ± 20	0.44	68.6 ± 25	83.2 ± 12.1	**0.016**

In bold - statistically significant result

On comparison of the thermometer rating (Table 3), representing overall health state as assessed subjectively by the patients, scores were similar in both groups at time0 (72.6 ± 22 vs. 68.6 ± 20, *p* > 0.05). In line with other reported QoL assessments, DJS patients score deteriorated over time, and they had a significant lower score compared to PCN patients at time1 (68.6 ± 25 vs. 83.2 ± 12.1 respectively, *p* < 0.05). On univariate analysis, drainage by PCN method, higher age, and decreased length of time to definitive procedure, were associated (all p < 0.05) with higher thermometer rating score at time1 (measured at presentation for definitive procedure). BMI, gender, mean thermometer score at time0, post-procedural pain, previous endourological procedure, stone load, and length of hospitalization were not associated with overall health score at time1. On multivariate analysis, no variable remained significantly associated with time1 overall health score.

## Discussion

Urgent decompression of obstructed collecting systems due to a ureteral calculus is a daily practice in every urology department in cases of infection, renal failure or intractable pain. Despite the commonness of this situation, there are only a few studies comparing renal drainage methods, including clinical and QoL aspects [1–3, 9, 10]. This study prospectively compared 45 DJS procedures to 30 PCN procedures. Selection criteria ensured that all patients were potential candidates for both procedures, and analysis revealed similar patients’ characteristics in both groups. QoL was evaluated with EuroQol EQ-5D and a ‘Tube symptoms’ questionnaire at two time points, in order to evaluate symptoms dynamics over time.

### Clinical course

In face of opposing results in former studies [1, 2], we found no distinct difference in post-procedural in-hospital pain, with patients in both groups reporting low VAS scores. The finding of higher post-operative pain for DJS in Mokhmalji et al. study [2] might be explained by the fact that retrograde placement was achieved by using a rigid cystoscope with prior transurethral administration of local anesthetic sedation, and had a 80% success rate. Procedure complication rate was also comparable between the methods, and consistent with other reports [11]. Ramsey at el. [11] reported in their review that there appears little evidence to suggest that retrograde stent insertion leads to increased bacteremia or is significantly more hazardous in the setting of acute obstruction. In league with this report, few patients at each group developed post drainage sepsis, with no clear advantage for any method. Moreover, post procedural recuperation was equivalent in both groups, as supported by similar time to defeverence and time of leukocytosis returning to normal range. However, it should be mentioned that we excluded patients presenting with septic complications or shock, which may benefit from PCN drainage in comparison to DJS. Although time of eGFR return to baseline was found to be longer in the PCN group vs. the DJS group (2 days vs. 1 day respectively), this finding could be explained by the lower baseline eGFR of the PCN group, and is probably not clinically significant. The longer hospitalization is probably associated with a slower recovery to baseline GFR in the PCN group, as patients were kept under observation to assess the kidney recovery. The apparent difference in time to second procedure was also noted in other studies [2]. DJS patients were summoned for their definitive treatment approximately twice as long as the nephrostomy patients. This substantial difference can be explained to some extent by a misguided perception that a patient with a DJS is less debilitated compared to a patient with a nephrostomy tube (an apparent external tube). Overall, our study supports similar clinical outcomes in both drainage methods.

### Quality of life

QoL outcomes in our study corresponded well with the only other prospective study comparing QoL at two time points after urgent renal drainage [2]. Both procedures caused pain or discomfort to a significant amount of patients. While the number of PCN patients complaining of pain remained similar over time, and analgesic use even lowered, more patients in the DJS group reported of pain, and analgesics use grew in prevalence and frequency as well. Clearly, the most troublesome symptoms in the DJS group are the urinary bother symptoms [3, 12]. These symptoms inflicted 80% of the patients in our DJS study group, much more frequent compared to other symptoms. Furthermore, there was no alleviation over time, the prevalence and severity of these symptoms did not change, translating to a higher number of emergency room visits. In distinct opposition, PCN patients suffered at first mostly from discomfort involving “movement”, “self-care” and “personal hygiene”. However, over time, these patients adjusted to the nephrostomy tube. The number of patients experiencing symptoms in these domains dropped by half, or more, reaching similar discomfort level with the DJS group at time1. These trends are manifested in the overall health state results. At first, both groups suffered from bothering symptoms, albeit different, amounting to similar overall disturbance. This result is corroborated by the recent study of de Sousa Morais N. et al [3]. Over time, PCN patients improved their symptoms, while DJS patients suffered similarly or worsened. This was reflected by significantly higher overall health state scores in the PCN group at time1. Of note, studies comparing PCN drainage vs DJS in other clinical scenarios [4, 5], such as following PCNL, also found that patients with DJS suffered significantly from pain and irritative symptoms that decreased the overall QoL. Interestingly, in the DJS group women reported higher level of pain / discomfort at both time points, and higher use of pain medications post operatively. In the PCN group, higher use of pain medications post operatively in women was also noted.

Is there a preferred approach for urgent decompression of obstructed collecting systems?

The decision on the appropriate method of drainage is multifactorial, including factors such as stone parameters, patient’s characteristics, patient’s and urologist preferences, the expected definitive approach for stone treatment and procedure availability. In their summary of 15 years outcomes, Goldsmith et al. [10] reported a tendency to prefer nephrostomy drainage in patients with larger stones and patients who are more acutely ill. Often, clinical judgment is exercised, and opinions may vary considerably [13]. When both procedures are available, and the patient is a potential candidate for either procedure, the expected clinical benefit and the expected QoL are major determinants in the decision. It appears that both methods result in similar good clinical result, with no significant benefit for the one over the other. Thus, the implications of the drainage on patient’s QoL should not be overlooked or disregarded, but rather become a factor in the decision on the preferred rout. A thorough discussion with the patient is extremely important, explaining the pros and cons of each procedure, not only clinically, but of what is expected regarding tube symptoms and QoL. In general, for proximal ureteral stones the advantages of pre-stenting probably outweigh the discomfort associated with the stent. However, for patients with a distal ureteral stone requiring drainage, a nephrostomy tube may be the best choice as it would allow possible spontaneous passage and would avoid the stent discomfort.

There are several limitations to our study. The “tube symptoms” questionnaire was based on a validated DJS symptoms questionnaire, adjusted to be relevant for both DJS and PCN groups, but was not validated in itself or to local language, and might induce recall bias. As both hospitals are public, at no cost to the patient, procedural or hospitalization cost did not introduce bias. However, selection bias may have been introduced through choice of drainage procedure according to surgeon’s preference, recruitment rate, possibly influenced by severity of eGFR, hydronephrosis, or other unmeasured parameters. Therefore patients suspected of long standing impaction probably had a higher chance of receiving a PCN, thus explaining the lower GFR in this group. Furthermore, as different imaging modalities were used, we could not assess hydronephrosis severity as a possible confounder. Finally, we did not assess a third time point, after tube removal, to confirm symptoms resolution. Nevertheless, we believe our study, supported by other past reports, demonstrate an overlooked truth. To quote Dr. Louis R Kavoussi [14], “Traditionally, urologists have placed stents because … that’s what we do”, but his experience is that “patients are more comfortable with a nephrostomy than a stent”. Interestingly, superior QoL of PCN over DJS placed after PCNL was also recently reported [4, 5]. The results of this study support superior QoL of nephrostomy tube over time, and may cause some urologists to reconsider their choice of renal drainage, especially in health systems in which definitive treatment might be delayed. Undoubtedly, the specific tube symptoms, and their influence of the patient over time, should be given a serious consideration in the deliberation for the right drainage method for a specific patient.

## Conclusions

No significant clinical difference was found in the outcomes and morbidity indices, in a prospective comparison between retrograde and antegrade renal drainage approaches in cases of acute ureteral obstruction by stone. The two techniques had a distinct and significantly different impact on quality of life. However, post-drainage symptoms improved with time only in patients treated with PCN. At the time of definite treatment, nephrostomy patients had significantly higher overall health state scores. Specific tube related symptoms, and their dynamics over time, should be a major determinant in choosing the appropriate drainage method, especially when definitive treatment is not imminent.

## Additional file

Additional file 1: Tube symptoms questionnaire. (DOCX 12 kb)

## Data Availability

The datasets used and/or analyzed during the current study are available from the corresponding author on reasonable request.

## Abbreviations

BMI: Body mass index
DJS: Ureteral double J stent
eGFR: Estimated glomerular filtration rate
PCN: Percutaneous nephrostomy tube
PCNL: Percutaneous nephrolithotomy
QoL: Quality of life
VAS: Visual analogue scale
