# Supplementary material for: Ureteric stent versus percutaneous nephrostomy for acute ureteral obstruction - clinical outcome and quality of life: a bi-center prospective study
Source: BMC Urol. 2019 Aug 28;19:79. doi: 10.1186/s12894-019-0510-4 (PMC6712738; doi:10.1186/s12894-019-0510-4)
Supplement: Supplementary file 1 — Tube symptoms questionnaire. (DOCX 12 kb) [file 12894_2019_510_MOESM1_ESM.docx]

**Tube symptoms questionnaire:**

|  | Never | Occasional | Frequent | Always |
| --- | --- | --- | --- | --- |
| How often do you feel discomfort or pain related to your tube? |  |  |  |  |
| How often do you need pain killers due to pain or discomfort related to your tube? |  |  |  |  |
| How often do you experience urination discomfort or pain? |  |  |  |  |
| How often do you experience blood in your urine? |  |  |  |  |
| How often do you experience personal hygiene problems due to your tube? |  |  |  |  |
| How often do feel that moving around is associated with discomfort? |  |  |  |  |
